# Clinical effects and safety of proximal femur bionic nail versus proximal femoral nail anti-rotation or InterTAN for the treatment of intertrochanteric femoral fracture: a systematic review and meta-analysis

**DOI:** 10.7717/peerj.20801

**Published:** 2026-02-16

**Authors:** Wenbin Zhang, Yulin Ma, Feilong Lu, Hao Song, Xiaoming Du, Zhaoxi Yang, Yimei Hu

**Affiliations:** 1 Qionglai Hospital of Traditional Chinese Medicine, Chengdu, China; 2 Affiliated Hospital of Chengdu University of Traditional Chinese Medicine, Chengdu, China

**Keywords:** Intertrochanteric femoral fracture, Proximal femur bionic nail, Proximal femoral nail anti-rotation, InterTAN, PFBN, PFNA, Meta-analysis

## Abstract

**Objective:**

To compare the clinical effects and safety of the proximal femur bionic nail (PFBN) with those of proximal femoral nail anti-rotation (PFNA) and InterTAN for the treatment of intertrochanteric femoral fracture (IFF).

**Methods:**

Studies comparing the clinical efficacy of PFBN with that of PFNA and InterTAN in the treatment of IFF published before 16 December 2025 in the PubMed, Embase, Web of Science, Cochrane, CNKI, Wanfang, and VIP databases were retrieved. After the research data were extracted, Review Manager 5.4 (RevMan 5.4 ) was used for data analysis.

**Results:**

A total of 15 studies involving 990 patients were included. The meta-analysis results indicated that the PFBN group had advantages over the control group in terms of the postoperative partial and full weight-bearing time, fracture healing time, fracture reduction quality, hospital stay, postoperative Harris score, intraoperative fluoroscopy time and postoperative complication rate (partial weight-bearing time: standardized mean difference (*SMD*) = −3.49, 95% confidence interval (CI) [−4.47 to −2.52], *P* < 0.00001; full weight-bearing time: *SMD* = −1.78, 95% CI [−2.86 to −0.70], *P* = 0.001; fracture healing time: *SMD* = −0.61, 95% CI −[0.86 to −0.37], *P* < 0.00001; fracture reduction quality: relative risk (*RR*) = 1.07, 95% CI [1.01∼1.13], *P* = 0.02; hospital stay: *SMD* = −0.44, 95% CI [−0.81 to −0.08], *P* = 0.02; postoperative complication rate: *RR* = 0.36, 95% CI [0.22∼0.59], *P* < 0.0001; postoperative Harris score: *SMD* = 0.32, 95% CI [0.04∼0.60], *P* = 0.02; intraoperative fluoroscopy time: *SMD* = 0.76, 95% CI [0.10∼1.42], *P* = 0.02). There was no significant difference between the two groups in terms of intraoperative blood loss, operation duration, postoperative hip range of motion, postoperative VAS score, or incision length (intraoperative blood loss: *SMD* = −0.30, 95% CI [−0.81∼0.21], *P* = 0.25; operation duration: *SMD* = 0.41, 95% CI [−0.03∼0.85], *P* = 0.07; postoperative flexion and extension motion: *SMD* = 0.28, 95% CI [−0.18∼0.73], *P* = 0.23; postoperative rotational motion: *SMD* = 0.20, 95% CI [−0.25∼0.66], *P* = 0.38; incision length: SMD = 0.23, 95% CI [−0.44∼0.89], *P* = 0.50; postoperative VAS score: *SMD* = −0.29, 95% CI [−0.82∼0.23], *P* = 0.27).

**Conclusion:**

For the treatment of IFF , the PFBN is more effective and has a lower risk than the PFNA and InterTAN.

## Introduction

Intertrochanteric femoral fracture (IFF) is among the most prevalent types of hip fractures. Projections suggest that by 2050, the global incidence of hip fractures will increase to 4.5–6 million annually, with IFF accounting for more than 50% of these cases (Chang et al., 2020; Veronese & Maggi, 2018). This condition tends to occur in elderly individuals and is characterized by a high rate of complications and mortality. Research indicates that the 6-month mortality rate for IFF in elderly patients is 7.6%, increasing to 13.9% within the first year and potentially reaching as high as 28.5% within 2 years (Wang et al., 2022b). Therefore, this disease is also known as the “last fracture of life”. With increasing age, its incidence increases annually (Wu et al., 2022). Currently, the primary treatment approaches for IFF are categorized into intramedullary and extramedullary fixation. Intramedullary fixation is usually the first choice for clinicians because it can minimize tissue damage and has high mechanical stability. It reduces tissue damage, significantly lowers intraoperative blood loss, and is conducive to the early recovery of patients (Xie et al., 2019; Zeelenberg et al., 2023). Coupled with high mechanical stability, it can promote early patient activities and reduce the occurrence of hip fracture complications (Zeelenberg et al., 2024). In hip fractures, intramedullary fixation can better resist strong bending stress (Lu, Li & Li, 2022). Compared with the lateral plate system, its mechanical failure rate and reoperation rate are significantly lower (Xie et al., 2019; Liu et al., 2015). This minimally invasive closed reduction and fixation technique maximizes the protection of blood supply to the fracture end, creating an excellent biological environment for bone healing, and ultimately translates into a better clinical prognosis and a lower incidence of functional disorders (Zhang et al., 2018). The primary methods for intramedullary fixation, including proximal femoral nail anti-rotation (PFNA), the Gamma nail, and InterTAN. However, although intramedullary nail fixation can significantly reduce tissue damage and provide high mechanical stability compared with extramedullary fixation, it still has deficiencies in aspects such as fracture reduction quality and the incidence of postoperative complications (Fragility Fracture Network-China et al., 2020; Chen et al., 2020a). These concerns are especially pertinent in the context of unstable fractures, such as the reverse-obliquity intertrochanteric fracture (Heydar & Kı yak, 2024; Mavrogenis et al., 2016; Li et al., 2019). The reason might be that the issue of tension loss after fracture has not been resolved (Zhu et al., 2021a). In view of these aspects, in recent years, many theories, such as “lever-balance-reconstruction” theory (Zhang et al., 2020; Zhang, 2020), “triangular support fixation” theory (Zhang et al., 2021c), “Zhang’s N-triangle” theory (Zhu et al., 2021a), and “triangular mechanical reconstruction of the proximal femur” (Zhang et al., 2021a), have been proposed to solve the current dilemma. In accordance with these theories, academician Zhang Yingze’s team and Professor Zhang Dianying’s team proposed the proximal femur bionic nail (PFBN) system (Zhang, Chen & Zhang, 2010; Zhang, Yu & Zhao, 2019). On the basis of these theories, PFBN adds a transverse interlocking support screw to the proximal end of the main nail, and the transverse screw, main nail and the anti-rotation screw form a stable triangular cross structure in the proximal femur, which significantly increases the stability and fixation strength of the proximal femur fracture. Enabling it to exert the greatest advantages in terms of anti-rotation, anti-pressure and anti-tension, and lowers complication rates. It is beneficial for fracture healing and weight-bearing activity (Chen et al., 2024; Wang et al., 2022c).

As a treatment strategy for IFF, PFBN is gaining attention for its ability to improve fracture reduction quality, promote healing, and enable early postoperative mobilization. Some clinical studies (Liu et al., 2023; Yang et al., 2023) highlight the potential clinical advantages of PFBN in promoting IFF healing and early activity without increasing the incidence of complications or surgical risks; however, this perspective lacks a comprehensive evaluation. Consequently, this study is the first comprehensive analysis of the clinical efficacy and safety of PFBN compared with PFNA and InterTAN in the treatment of IFF, aiming to provide evidence-based medical support for the clinical application of PFBN in IFF treatment.

## Materials and Methods

The study was conducted according to our preregistered protocol on PROSPERO and the guidance of the PRISMA statement (Page et al., 2021). The PROSPERO registration number for this study is **CRD42024549360**. Portions of this text were previously published as part of a preprint (https://www.researchsquare.com/article/rs-5336804/v1).

### Inclusion and exclusion criteria

#### Inclusion criteria

(1) Study type: clinical controlled study. (2) Study population: patients diagnosed with IFF requiring surgical treatment. (3) Interventions: the intervention group was treated with PFBN, whereas the control group was treated with PFNA or InterTAN. (4) Outcome: included at least one or more of the following: partial weight bearing time, full weight bearing time, fracture healing time, fracture reduction quality, Harris scale, hospital stay, operation duration, intraoperative blood loss, postoperative complication rate, femoral neck–shaft angle, tip–apex distance, hip range of motion, VAS scale, *etc*.

#### Exclusion criteria

(1) studies with incomplete data for analysis; (2) full texts not available; and (3) duplicate studies.

### Search strategy

A search was conducted in databases including PubMed, WOS, Embase, *Cochrane*, CNKI, Wanfang, and VIP for clinically controlled studies on the treatment of intertrochanteric femoral fracture (IFF) with PFBN published prior to 16 December 2025. “Proximal femur bionic nail” and “PFBN” were used as search terms. The search strategy is shown in Appendix S1.

### Literature screening and data extraction

After the studies were retrieved, they were imported into EndNote, and duplicate references were removed. Two researchers (Zhang and Ma) independently screened the literature on the basis of the inclusion and exclusion criteria. The titles and abstracts of the remaining studies were read after removing duplicates and preliminarily excluding irrelevant studies, after which the full texts of the remaining studies were read. Finally, data from the literature, including basic features of the literature (author names, country, publication year, *etc*.) and the patients (intervention and control measures, sample size, sex ratio, age, follow-up time, *etc*.), trial results, and quality assessment methods, were extracted. In the case of any disagreements, the final determination will be made by a senior researcher (Hu). If only the median (interquartile range) is given in the study, the conversion is made *via* online data conversion (https://www.math.hkbu.edu.hk/ tongt/papers/median2mean.html). After the extraction was complete, the data were entered into Review Manager 5.4 (RevMan 5.4) software (Cochrane Collaboration, 2020) for analysis.

### Quality evaluation of the literature

The quality of cohort studies was assessed according to the Newcastle-Ottawa Quality Assessment Scale (NOS) (Stang, 2010). A funnel plot was used to assess the presence of publication bias in the included studies.

### Statistical methods

Data analysis was performed *via* RevMan 5.4. Binary variables are expressed as the relative risk (*RR*) and 95% confidence interval (*CI*), whereas continuous variables are expressed as the standardized mean difference (*SMD*) and 95% CI. For heterogeneity testing, the chi-square test and *I^2^* test were used. When *I^2^* ≤ 50% and *P* ≥ 0.1, low heterogeneity was indicated, and a fixed effects model was selected. Conversely, if *I^2^* > 50% or *P* < 0.1, high heterogeneity was suggested. If the source of heterogeneity could not be identified, a random effects model was chosen for analysis, subgroup analysis and sensitivity analysis was conducted when necessary. A difference was considered statistically significant when *P* < 0.05.

## Results

### Literature search results

**Figure 1:**
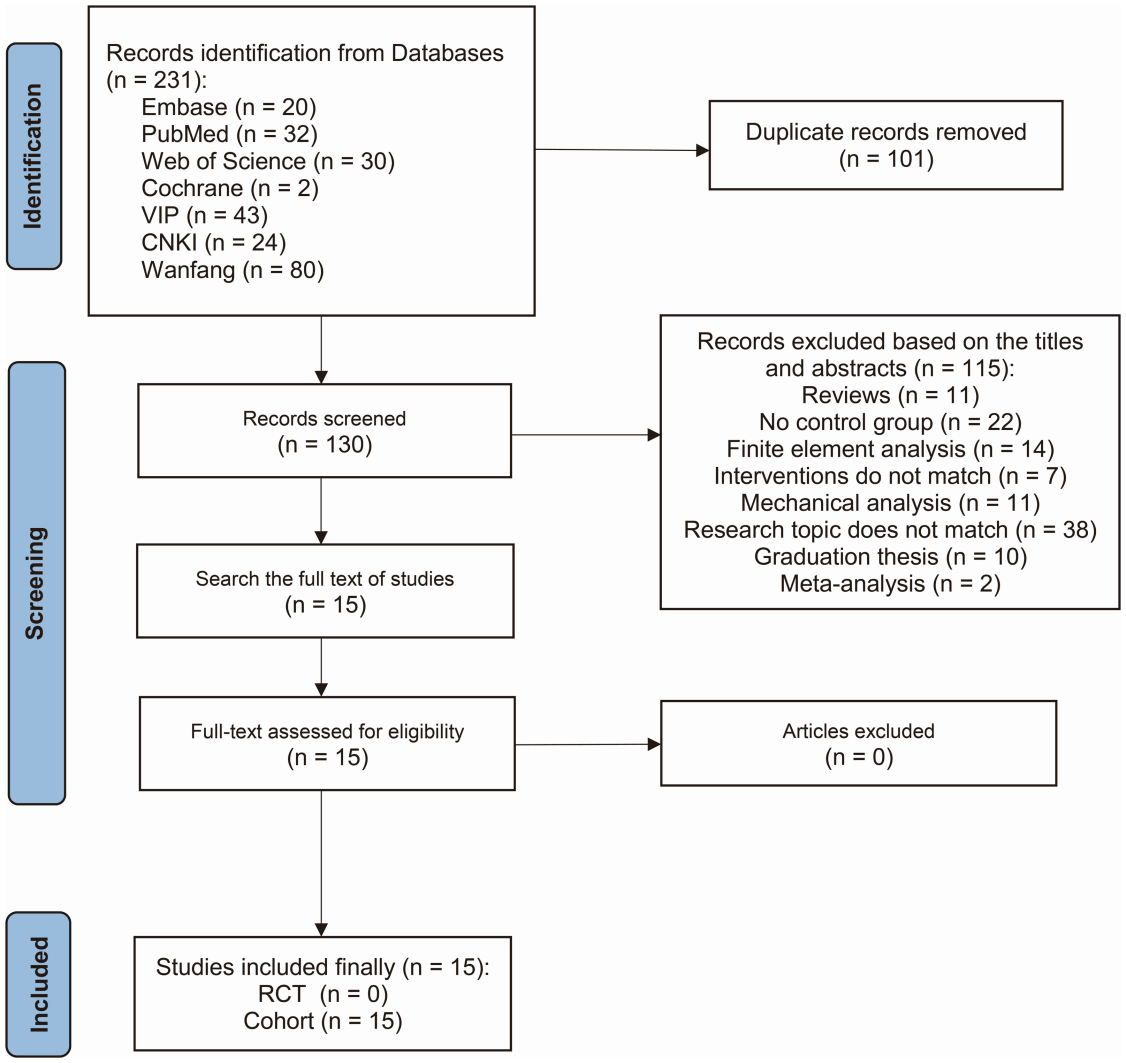
Flow chart of the retrieval strategy.

In the initial assessment, 231 studies were identified. Following a rigorous preliminary review, the full-text examination led to the retention of 15 studies that met the inclusion criteria of this research (Liu et al., 2023; Yang et al., 2023; Fu et al., 2023; Jin et al., 2024; Long & Li, 2022; Ling et al., 2023; Li et al., 2022; Lin et al., 2022; Zhang & Peng, 2022; Wang et al., 2023; Wu et al., 2023; Wan et al., 2024; Jia et al., 2023; Fu et al., 2024; Zhang, Zhang & Zhang, 2024). Notably, all 15 retained studies had a cohort design. The literature screening process is depicted in Fig. 1.

A total of 15 studies with 990 patients were included, of which 401 were treated with PFBN, 476 with PFNA, and 113 with InterTAN. The essential characteristics of the studies included in this study are provided in Table 1.

**Table 1:** Basic characteristics of the included studies.

Author (Year)	Group	Sample size (F/M)	Mean age (years old)	Outcome	NOS
Fu et al. 2023)	PFBN PFNA InterTAN	18 (10/8) 36 (24/12) 14 (9/5)	76.0 ± 4.8 79.7 ± 7.8 81.4 ± 7.6	(1)(3)(5)(10)	9
Jin et al. (2024)	PFBN PFNA InterTAN	25 (16/9) 55 (38/17) 40 (26/14)	73.67 ± 5.16 74.23 ± 5.57 73.45 ± 5.34	(1)(3)(5)(7)(8)(9)(10)(15)	9
Liu et al. (2023)	PFBN PFNA	35 (15/20) 37 (15/22)	71.81 ± 7.63 72.33 ± 6.47	(1)(2)(3)(4)(5)(7)(8)(9)(10)(14)(15)	8
Long & Li (2022)	PFBN PFNA	20 (9/11) 60 (21/39)	63.57 ± 6.84 63.29 ± 5.84	(1)(3)(4)(5)(7)(8)(9)(10)	8
Ling et al. (2023)	PFBN PFNA	28 (16/12) 28 (18/10)	70.4 ± 7.8 73.0 ± 8.9	(3)(4)(5)(7)(9)(10)	8
Li et al. (2022)	PFBN PFNA	46 (24/22) 46 (26/20)	75.7 ± 5.2 75.3 ± 4.2	(1)(4)(5)(7)(8)(9)(10)	8
Lin et al. (2022)	PFBN PFNA	20 (14/6) 25 (18/7)	78.7 ± 5.9 78.7 ± 8.2	(2)(3)(5)(7)(9)(10)(12)(13)	9
Zhang & Peng (2022)	PFBN PFNA	32 (13/19) 32 (12/20)	79.24 ± 1.23 79.17 ± 1.21	(1)(8)(9)(10)	7
Yang et al. (2023)	PFBN PFNA	24 (13/11) 24 (15/9)	79.0 ± 5.0 78.6 ± 5.8	(1)(3)(4)(6)(7)(8)(9)(10)	8
Wang et al. (2023)	PFBN PFNA	20 (11/9) 20 (12/8)	75.3 ± 6.4 74.6 ± 6.0	(1)(2)(5)(6)(8)(9)(10)(11)(12)(15)	8
Wu et al. (2023)	PFBN PFNA	30 (13/17) 30 (12/18)	69.4 ± 3.5 69.6 ± 3.2	(3)(5)(7)(9)(10)	8
Wan et al. (2024)	PFBN InterTAN	16 (11/5) 19 (15/4)	78.0 ± 8.8 75.3 ± 7.0	(1)(2)(5)(6)(8)(9)(10)(11)(13)(14)(15)	9
Jia et al. (2023)	PFBN InterTAN	25 (17/8) 20 (13/7)	83.28 ± 7.85 79.90 ± 9.60	(1)(2)(3)(4)(5)(7)(8)(9)(10)(14)	9
Fu et al. (2024)	PFBN PFNA InterTAN	22 (12/20) 40 (27/13) 20 (13/7)	76.27 ± 4.47 79.15 ± 7.82 80.55 ± 7.05	(1)(3)(7)(8)(9)(10)(14)	8
Zhang, Zhang & Zhang (2024)	PFBN PFNA	40 (25/15) 43 (27/16)	81.4 ± 9.1 80.2 ± 11.6	(5)(6)(7)(8)(9)(10)	9

**Notes.**

(1) Partial weight bearing time; (2) Full weight bearing time; (3) Fracture healing time; (4) Fracture reduction quality; (5) Harris scale; (6) VAS; (7) Postoperative complication rate; (8) Hospital stay; (9) Intraoperative blood loss; (10) Operation duration; (11) Hip range of motion; (12) Femoral neck-shaft angle; (13) Tip-apex distance; (14) Intraoperative fluoroscopy times; (15) Incision length.

### Quality assessment results

Among the included cohort studies, exposure determination and cohort selection were distinct and comparable, with no pre-existing outcomes. The outcome index measurement method was suitable. Eight studies (Liu et al., 2023; Yang et al., 2023; Long & Li, 2022; Ling et al., 2023; Li et al., 2022; Wang et al., 2023; Wu et al., 2023; Fu et al., 2024) reported follow-up periods of less than one year. One study (Zhang & Peng, 2022) did not detail the follow-up times or procedures and thus was not scored. All studies scored over seven on the NOS scale, ensuring quality.

### Meta-analysis results

A summary of the meta-analysis results is shown in Table 2.

**Table 2:** Summary of the meta-analysis results of the included studies.

Outcome	Subgroup	Heterogeneity test		Effect models	Meta-analysis results		
		*I* ^2^	*P*		*SMD/RR*	95% CI	*P*
Partial weight bearing time^a^	PFNA	94%	<0.00001	R	−3.60	[−4.70, −2.51]	<0.00001
	InterTAN	96%	<0.00001		−3.20	[−5.62, −0.79]	0.0009
	Overall	95%	<0.00001		−3.49	[−4.47, −2.52]	<0.00001
Full weight bearing time^a^	PFNA	91%	<0.0001	R	−1.61	[−2.88, −0.33]	0.01
	InterTAN	/	/		−2.34	[−3.23, −1.46]	<0.00001
	Overall	89%	<0.00001		−1.78	[−2.86, −0.70]	0.001
Fracture healing time^a^	PFNA	74%	<0.0001	R	−0.56	[−0.89, −0.23]	0.0008
	InterTAN	0%	0.90		−0.76	[−1.06, −0.46]	<0.00001
	Overall	65%	0.0004		−0.61	[−0.86, −0.37]	<0.00001
Fracture reduction quality^a^	PFNA	65%	0.01	R	1.08	[1.01, 1.16]	0.02
	InterTAN	0%	0.56		1.01	[0.89, 1.15]	0.87^*^
	Overall	47%	0.07		1.07	[1.01, 1.13]	0.02
Harris scale^a^	PFNA	66%	0.007	R	0.41	[0.07, 0.75]	0.02
	InterTAN	51%	0.13		0.10	[−0.38, 0.59]	0.68^*^
	Overall	63%	0.003		0.32	[0.04, 0.60]	0.02
VAS scale^a^	PFNA	80%	0.007	R	−0.34	[−1.05, 0.37]	0.35^*^
	InterTAN	/	/		−0.18	[−0.84, 0.49]	0.60^*^
	Overall	70%	0.02		−0.29	[−0.82, 0.23]	0.27^*^
Postoperative complication rate	PFNA	0%	1.00	F	0.32	[0.18, 0.56]	<0.0001
	InterTAN	0%	0.95		0.58	[0.22, 1.54]	0.28^*^
	Overall	0%	1.00		0.36	[0.22, 0.59]	<0.0001
Hospital stay^a^	PFNA	80%	<0.00001	R	−0.33	[−0.70, 0.05]	0.09^*^
	InterTAN	90%	<0.00001		−0.77	[−1.78, 0.24]	0.14^*^
	Overall	83%	<0.00001		−0.44	[−0.81, −0.08]	0.02
Intraoperative blood loss^a^	PFNA	94%	<0.00001	R	−0.52	[−1.16, 0.12]	0.11^*^
	InterTAN	80%	0.002		0.30	[−0.37, 0.98]	0.38^*^
	Overall	93%	<0.00001		−0.30	[−0.81, 0.21]	0.25^*^
Operation duration^a^	PFNA	92%	<0.00001	R	0.32	[−0.22, 0.86]	0.24^*^
	InterTAN	87%	<0.00001		0.65	[−0.14, 1.45]	0.11^*^
	Overall	91%	<0.00001		0.41	[−0.03, 0.75]	0.07^*^
Hip range of motion	Flexion and extension	0%	0.53	F	0.28	[−0.18, 0.73]	0.23^*^
	Rotation	0%	0.8		0.2	[−0.25, 0.66]	0.38^*^
Femoral neck-shaft angle^a^	PFNA	78%	0.03	R	−0.01	[−0.93, 0.92]	0.99^*^
	Overall	67%	0.03		0.32	[−0.23, 0.87]	0.25^*^
Tip-apex distance	PFNA	/	/	F	−0.49	[−1.08, 0.11]	0.11^*^
	InterTAN	/	/		−0.9	[−1.61, −0.20]	0.01
	Overall	0%	0.37		−0.66	[−1.12, −0.21]	0.004
Intraoperative fluoroscopy times^a^	PFNA	89%	0.0001	R	0.75	[−0.23, 1.72]	0.13^*^
	InterTAN	89%	0.0002		0.80	[−0.35, 1.95]	0.17^*^
	Overall	86%	<0.00001		0.76	[0.10, 1.42]	0.02
Incision length^a^	PFNA	89%	0.0001	R	0.26	[−0.67, 1.19]	0.59^*^
	InterTAN	91%	0.001		0.21	[−1.23, 1.65]	0.78^*^
	Overall	86%	<0.00001		0.23	[−0.44, 0.89]	0.50^*^

**Notes.**

a The results of the sensitivity analysis were stable, and the results of the random effects analysis were acceptable.

* No significant difference between the two groups.

R, Random effects model; F, Fixed effects model.

#### Partial weight bearing time

**Figure 2:**
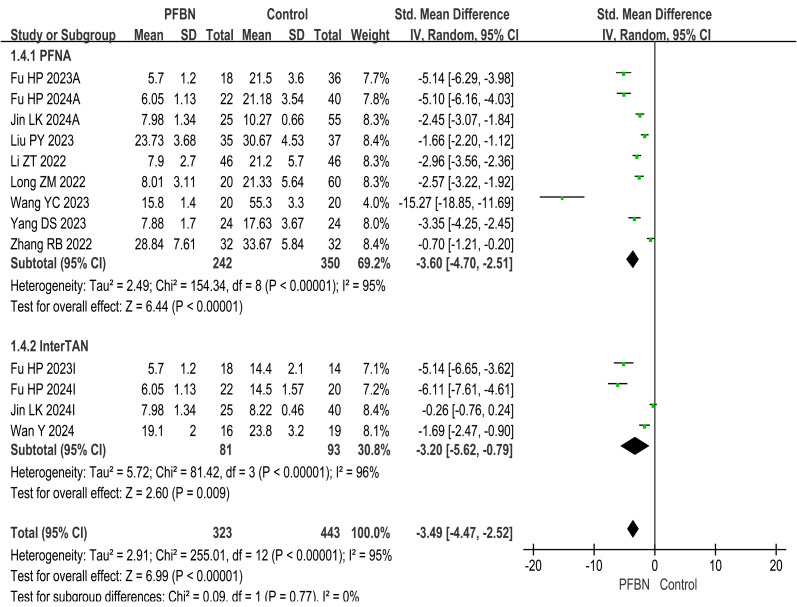
Meta-analysis results of partial weight-bearing time after surgery.

A total of 10 studies (Liu et al., 2023; Yang et al., 2023; Fu et al., 2023; Jin et al., 2024; Long & Li, 2022; Li et al., 2022; Zhang & Peng, 2022; Wang et al., 2023; Wan et al., 2024; Fu et al., 2024) reported partial weight-bearing time after surgery. Considerable heterogeneity was observed across the studies. As sensitivity analyses (*e.g.*, leave-one-out method) failed to pinpoint a specific source of this heterogeneity, a random-effects model was therefore applied for the meta-analysis. The results revealed that the partial weight-bearing time in the PFBN group was significantly shorter than that in the control group (PFNA: *SMD* = −3.60, 95% CI [−4.70∼−2.51], *P* < 0.00001; InterTAN: *SMD* = −3.20, 95% CI [−5.62∼−0.79], *P* = 0.009; Overall: *SMD* = −3.49, 95% CI [−4.47∼−2.52], *P* < 0.00001). As shown in Table 2 and Fig. 2.

#### Full weight bearing time

**Figure 3:**
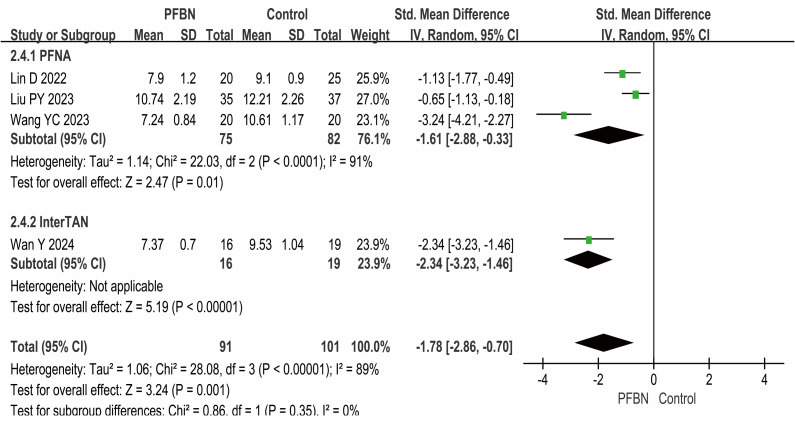
Meta-analysis results of full weight-bearing time after surgery.

Four studies (Yang et al., 2023; Lin et al., 2022; Wang et al., 2023; Wan et al., 2024) reported the full weight-bearing time after surgery, with the PFBN group showing a significantly shorter duration than the control group did (PFNA: *SMD* = −1.61, 95% CI [−2.88∼−0.33], *P* = 0.01; InterTAN: *SMD* = −2.34, 95% CI [−3.23∼−1.46], *P* < 0.00001; Overall: *SMD* = −1.78, 95% CI [−2.86∼−0.70], *P* = 0.001). Considerable heterogeneity was observed across the studies. As sensitivity analyses (*e.g.*, leave-one-out method) failed to pinpoint a specific source of this heterogeneity, a random-effects model was therefore applied for the meta-analysis. As shown in Table 2 and Fig. 3.

#### Fracture healing time

**Figure 4:**
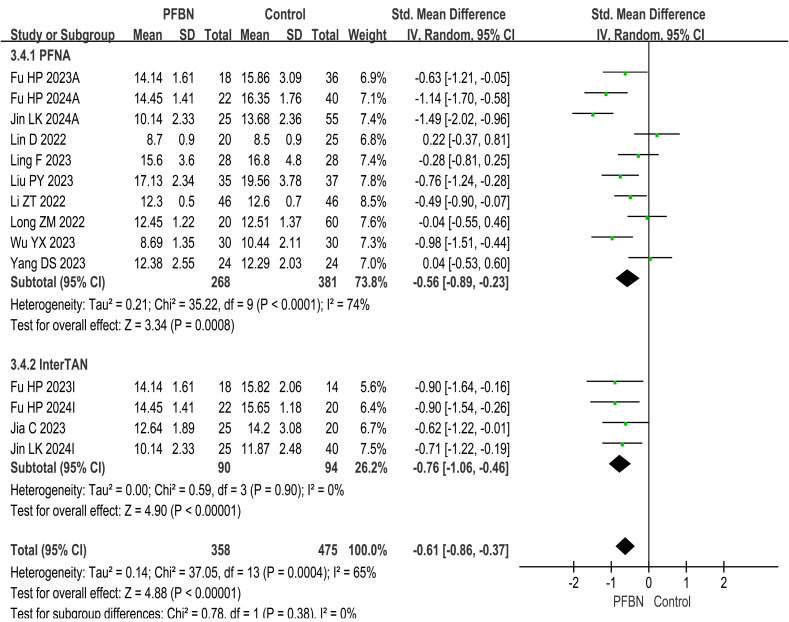
Meta-analysis results of fracture healing time.

A total of 11 studies (Liu et al., 2023; Yang et al., 2023; Fu et al., 2023; Jin et al., 2024; Long & Li, 2022; Ling et al., 2023; Li et al., 2022; Lin et al., 2022; Wu et al., 2023; Jia et al., 2023; Fu et al., 2024) reported fracture healing times. Considerable heterogeneity was observed across the studies. As sensitivity analyses (*e.g.*, leave-one-out method) failed to pinpoint a specific source of this heterogeneity, a random-effects model was therefore applied for the meta-analysis. The results revealed that the fracture healing time in the PFBN group was significantly shorter than that in the control group (PFNA: *SMD* = −0.56 95% CI [−0.89∼−0.23], *P* = 0.0008; InterTAN: *SMD* = −0.76, 95% CI [−1.06∼−0.46], *P* < 0.00001; Overall: *SMD* = −0.61, 95% CI [−0.86∼−0.37], *P* < 0.00001). As shown in Table 2 and Fig. 4.

#### Postoperative complication rate (safety assessment)

**Figure 5:**
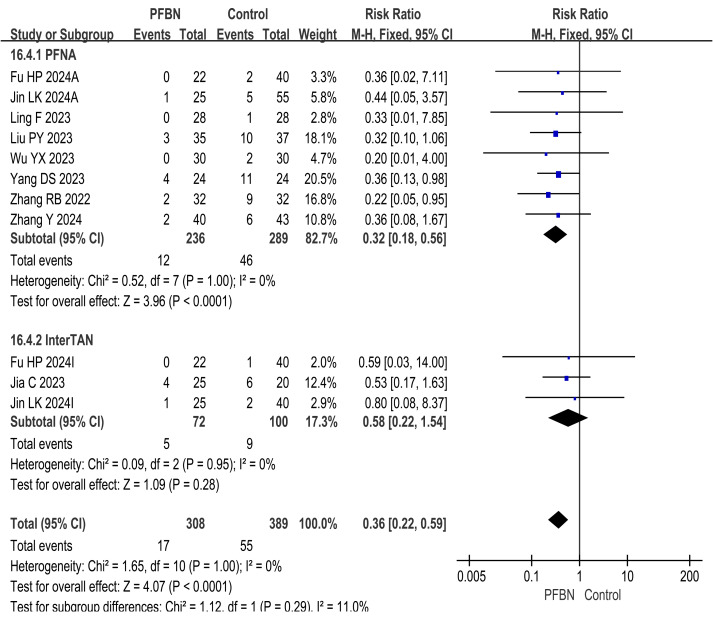
Meta-analysis results of the postoperative complication rate.

A total of nine studies (Liu et al., 2023; Yang et al., 2023; Jin et al., 2024; Ling et al., 2023; Zhang & Peng, 2022; Wu et al., 2023; Jia et al., 2023; Fu et al., 2024; Zhang, Zhang & Zhang, 2024) reported postoperative complication rates, known as safety assessments. The heterogeneity among the studies was relatively small, and a fixed-effect model was used for analysis. The results revealed that the postoperative complication rate in the PFBN group was significantly lower than that in the control group (PFNA: *RR* = 0.32, 95% CI [0.18∼0.56], *P* < 0.0001; InterTAN: *RR* = 0.58, 95% CI [0.22∼1.54], *P* = 0.28; Overall: *RR* = 0.36, 95% CI [0.22∼0.59], *P* < 0.0001). Thus, to a certain extent, patients with IFF are relatively safer in choosing PFBN treatment. As shown in Table 2 and Fig. 5.

#### Fracture reduction quality

**Figure 6:**
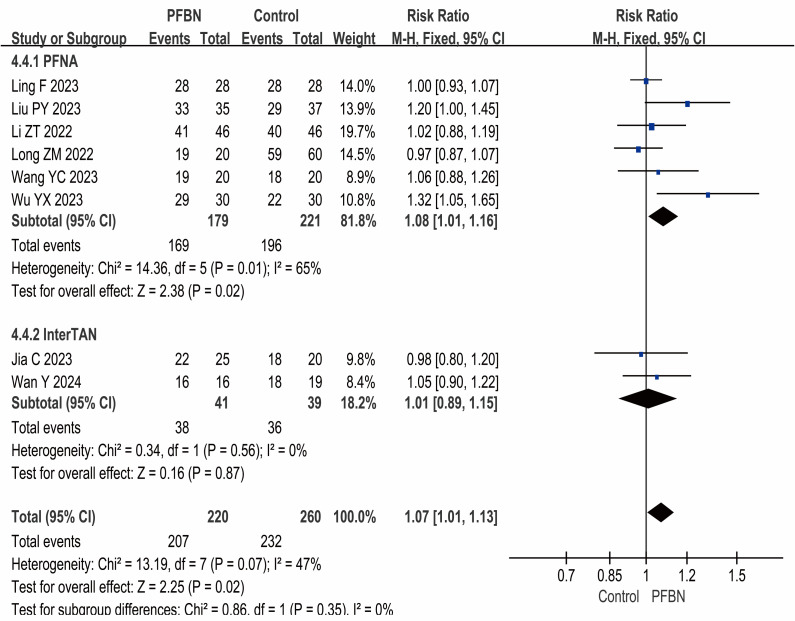
Meta-analysis results of fracture reduction quality.

Eight studies (Liu et al., 2023; Long & Li, 2022; Ling et al., 2023; Li et al., 2022; Wang et al., 2023; Wu et al., 2023; Wan et al., 2024; Jia et al., 2023) reported the fracture reduction quality, that is, the rates of good and excellent reduction quality. The heterogeneity among the studies was relatively small, and a fixed-effect model was used for analysis. The results revealed that the quality of fracture reduction in the PFBN group was greater than that in the control group, and the difference was statistically significant (PFNA: *RR* = 1.08, 95% CI [1.01∼1.16], *P* = 0.02; InterTAN: *RR* = 1.01, 95% CI [0.89∼1.15], *P* = 0.87; Overall: *RR* = 1.07, 95% CI [1.01∼1.13], *P* = 0.02). As shown in Table 2 and Fig. 6.

#### Postoperative hip Harris score

Nine studies (Liu et al., 2023; Jin et al., 2024; Ling et al., 2023; Lin et al., 2022; Wang et al., 2023; Wu et al., 2023; Wan et al., 2024; Jia et al., 2023; Zhang, Zhang & Zhang, 2024) reported postoperative hip Harris scores. Considerable heterogeneity was observed across the studies. As sensitivity analyses (*e.g.*, leave-one-out method) failed to pinpoint a specific source of this heterogeneity, a random-effects model was therefore applied for the meta-analysis. The score in the control group was lower than that in the PFBN group, and the difference was statistically significant (PFNA: *SMD* = 0.41, 95% CI [0.07∼0.75], *P* = 0.02; InterTAN: *SMD* = 0.10, 95% CI [−0.38∼0.59], *P* = 0.68; Overall: *SMD* = 0.32, 95% CI [0.04∼0.60], *P* = 0.02). As shown in Table 2 and Fig. S1.

#### Postoperative VAS score

A total of four studies (Yang et al., 2023; Wang et al., 2023; Wan et al., 2024; Zhang, Zhang & Zhang, 2024) reported postoperative VAS scores. Considerable heterogeneity was observed across the studies. Sensitivity analysis using the leave-one-out method indicated that the heterogeneity changed significantly upon the exclusion of the study by Zhang, Zhang & Zhang (2024) alone. The results showed that the PFBN group had lower postoperative VAS scores compared to the control group, but the difference was not statistically significant (PFNA: *SMD* = −0.34, 95% CI [−1.05∼0.37], *P* = 0.35; InterTAN: *SMD* = −0.18, 95% CI [−0.84∼0.49], *P* = 0.60; Overall: *SMD* = −0.29, 95% CI [−0.82∼0.23], *P* = 0.27). As shown in Table 2 and Fig. S2.

#### Hospital stay

A total of 11 studies (Liu et al., 2023; Yang et al., 2023; Jin et al., 2024; Long & Li, 2022; Li et al., 2022; Zhang & Peng, 2022; Wang et al., 2023; Wan et al., 2024; Jia et al., 2023; Fu et al., 2024; Zhang, Zhang & Zhang, 2024) reported the duration of hospital stay. Considerable heterogeneity was observed across the studies. As sensitivity analyses (*e.g.*, leave-one-out method) failed to pinpoint a specific source of this heterogeneity, a random-effects model was therefore applied for the meta-analysis. The results indicated that while the PFBN group had shorter hospital stays than did the control group did, the difference was statistically significant (PFNA: *SMD* = −0.33, 95% CI [−0.70∼0.05], *P* = 0.09; InterTAN: *SMD* = −0.77, 95% CI [−1.78∼0.24], *P* = 0.31; Overall: *SMD* = −0.44, 95% CI [−0.81∼−0.08], *P* = 0.02). As shown in Table 2 and Fig. S3.

#### Intraoperative blood loss

Fourteen studies (Liu et al., 2023; Yang et al., 2023; Jin et al., 2024; Long & Li, 2022; Ling et al., 2023; Li et al., 2022; Lin et al., 2022; Zhang & Peng, 2022; Wang et al., 2023; Wu et al., 2023; Wan et al., 2024; Jia et al., 2023; Fu et al., 2024; Zhang, Zhang & Zhang, 2024) reported intraoperative blood loss. Considerable heterogeneity was observed across the studies. As sensitivity analyses (*e.g.*, leave-one-out method) failed to pinpoint a specific source of this heterogeneity, a random-effects model was therefore applied for the meta-analysis. The findings revealed that there is no statistically significant difference in intraoperative blood loss between the PFBN group and the control group (PFNA: *SMD* = −0.52, 95% CI [−1.16∼0.12], *P* = 0.11; InterTAN: *SMD* = 0.30, 95% CI [−0.37∼0.98], *P* = 0.38; Overall: *SMD* = −0.30, 95% CI [−0.81∼0.21], *P* = 0.25). As shown in Table 2 and Fig. S4.

#### Operation duration

A total of 15 studies (Liu et al., 2023; Yang et al., 2023; Fu et al., 2023; Jin et al., 2024; Long & Li, 2022; Ling et al., 2023; Li et al., 2022; Lin et al., 2022; Zhang & Peng, 2022; Wang et al., 2023; Wu et al., 2023; Wan et al., 2024; Jia et al., 2023; Fu et al., 2024; Zhang, Zhang & Zhang, 2024) reported the operation duration. Considerable heterogeneity was observed across the studies. As sensitivity analyses (*e.g.*, leave-one-out method) failed to pinpoint a specific source of this heterogeneity, a random-effects model was therefore applied for the meta-analysis. Although the operation duration in the control group was shorter than that in the PFBN group, the difference was not statistically significant (PFNA: *SMD* = 0.32, 95% CI [−0.22∼0.86], *P* = 0.24; InterTAN: *SMD* = 0.65, 95% CI [−0.14∼1.45], *P* = 0.11; Overall: *SMD* = 0.41, 95% CI [−0.03∼0.85], *P* = 0.07). As shown in Table 2 and Fig. S5.

#### Other indicators

In terms of other indicators, the analysis results revealed that, except for tip−apex distance (*SMD* = −0.66, 95% CI [−1.12∼−0.21], *P* = 0.0004) and intraoperative fluoroscopy time (*SMD* = 0.76, 95% CI [0.10∼1.42], *P* = 0.02), there were no significant differences between the PFBN group and the control group in terms of postoperative hip range of motion, femoral neck−shaft angle, or incision length (flexion and extension: *SMD* = 0.28, 95% CI [−0.18∼0.73], *P* = 0.23; rotation: *SMD* = 0.20, 95% CI [−0.25∼0.66], *P* = 0.38; femoral neck−shaft angle: *SMD* = 0.32, 95% CI [−0.23∼0.87], *P* = 0.25; incision length: SMD = 0.23, 95% CI [−0.44∼0.89], *P* = 0.50). As shown in Table 2.

### Sensitivity analysis

In this study, sensitivity analysis was carried out on the above studies with high heterogeneity *via* leave-one-out and change effect models. The results revealed that all the results were relatively stable and reliable with no significant changes, and no source of heterogeneity was found. The analysis results of the random effects model were acceptable.

### Publication bias

The operation duration was used as an example to assess publication bias. The funnel plot, which displays a symmetrical funnel shape, indicates that the risk of publication bias in the included studies was not significant. As shown in Fig. S6.

## Discussion

### Findings of this study

This study conducted a systematic review and meta-analysis of the clinical efficacy and safety of PFBN compared with PFNA or InterTAN. Compared with previous related research findings, the following characteristics are presented:

In terms of clinical efficacy, this study indicates that PFBN may have certain advantages in some aspects. Compared with PFNA and InterTAN, PFBN demonstrates superior performance in maintaining the stability of fracture reduction and reducing the time required for fracture healing. It allows for earlier ambulation postoperatively without increasing the risk of internal fixation failure (Atzmon et al., 2022; Jia et al., 2021). This not only aids in patients’ quicker recovery of physical functions and enhances their independence and autonomy in daily life but also has a positive and profound impact on the overall rehabilitation process. These benefits include the prevention of complications and a reduction in mortality rates (Atzmon et al. 2022). This may be attributed to its unique biomimetic design, which better conforms to the anatomical structure of the proximal femur, restores the physiological fulcrum position of the proximal femur, provides a more appropriate stress distribution, and offers more sustained and robust fixation (Wang et al., 2022c; Wang et al., 2022a; Cheng et al., 2023). PFNA and InterTAN may not provide ideal fixation for the lateral wall of the IFF, and the spiral blade of its anti-rotating screw is not supported effectively, which may result in a significant increase in cutting stress. This can result in displacement of the femoral head, followed by the four major challenges of ”retrograde nail, rotation, cutting, and instability”. Subsequently, complications such as failure of internal fixation and varus collapse of the hip may arise (Mavrogenis et al., 2016; Nie et al., 2021; Zhang et al., 2021b; Shi et al., 2021). Since these shortcomings were identified, efforts have been continuously made to explore solutions (Wu et al., 2022; Wang et al., 2022a; Chen et al., 2020b; Chen et al., 2023).

In terms of safety, the amount of intraoperative blood loss in PFBN is relatively lower than that in PFNA, while there is no significant difference compared with that in InterTAN. The incidence of postoperative complications also exhibited the same characteristics. Some studies have also indicated that PFBN has a lower mortality rate than hip replacement does (Sun et al., 2024), suggesting that PFBN may have a potential advantage in reducing surgical risk. However, there is also complexity and specificity between different treatment methods, a characteristic that warrants in-depth analysis. PFBN may enhance internal fixation stability and reduce the risk of complications through its more optimized structural design. By triangulating the tension screws and pressure screws, the fractures of the main pressure bone trabeculae are repaired, the shear forces on the main tension bone trabeculae are reduced (Zhu et al., 2021a; Zhang et al., 2021c), and the center of the force arm is closer to the physiological fulcrum (Lin et al., 2022). This design aligns the forces more closely with the body’s natural mechanics, which can improve the stability of internal fixation and reduce the risk of complications such as loosening or failure of internal fixation (Zhu et al., 2021b). This not only reduces the intraoperative risk but also may have a positive impact on patients’ early postoperative recovery and reduce the incidence of complications. For example, it can lead to a decrease in anemia-related complications, accelerate postoperative weight bearing, reduce postoperative pain, and mitigate inflammatory responses.

Compared with PFNA and InterTAN, PFBN did not significantly differ in terms of surgical duration, incision length, or postoperative hip range of motion (Table 2). These findings suggest that PFBN does not have a clear advantage over PFNA or InterTAN in terms of certain surgical and postoperative recovery indicators and that the overall efficacy of the three internal fixation methods is essentially comparable. As seen from the forest plot, the operative times of PFNA and InterTAN were numerically shorter than that of PFBN, although the difference was not statistically significant. The reason might be that the insertion of transverse support screws has, to some extent, increased the surgical time (Fu et al., 2023; Jin et al., 2024; Long & Li, 2022). Additionally, the surgical duration and length of hospital stay may also vary due to the interactive effects of various other factors, such as the surgeon’s experience and technical skill, the patient’s physical condition, and postoperative care and rehabilitation management. These factors can significantly influence patient outcomes and should be taken into account when evaluating the overall surgical process and recovery. The length of the surgical incision is likely to be primarily determined by the choice of surgical approach rather than the internal fixation device itself. The number of intraoperative fluoroscopies is likely more closely related to the complexity of the surgery and the surgeon’s experience rather than being solely dependent on the type of internal fixation used. Postoperative hip range of motion is more closely associated with factors such as postoperative rehabilitation exercises.

In summary, PFBN has advantages in terms of early postoperative weight bearing time, fracture healing time, postoperative complication rates, length of hospital stay, and intraoperative blood loss. However, in other aspects, the differences between PFBN and PFNA or InterTAN are not significant. This provides an important reference for the selection of clinical treatment plans. On the basis of the patient’s specific conditions and the surgeon’s experience, the unique advantages of PFBN can be weighed against the relative consistency in other aspects to formulate the most appropriate surgical strategy.

### Limitations and strengths

#### Strengths of this study

Currently, only a limited number of observational studies on efficacy have been published, and their conclusions remain inconsistent. Notably, there is a lack of comprehensive meta-analyses that systematically synthesize the available evidence. On the basis of existing clinical studies, this study aims to perform a meta-analysis to evaluate the comparative efficacy and safety of PFBN *versus* PFNA and InterTAN in the treatment of IFF. The findings are expected to provide valuable evidence to inform clinical decision-making in IFF management, which also constitutes the strength and novelty of this work.

#### Limitations of this study

First, all the studies included in this paper were cohort studies, which are considered to have a lower level of evidence than randomized controlled trials (RCTs), this to some extent lowers the level of evidence in the article. Second, some studies have relatively short follow-up periods (less than 1 year), which lack long-term assessment and may have a certain impact on the evaluation of outcomes. Third, the sample sizes of the included studies were generally small, and large-sample RCTs to corroborate the findings are lacking. Fourth, owing to the variability in the proficiency of the surgical staff, there may be differences in the assessment of intraoperative indicators such as surgical time and intraoperative blood loss. Finally, the heterogeneity of the analysis results of most outcome indicators was relatively high, which to some extent reduced the evidence level of the research results. This study has standardized all the assessment criteria to minimize such impacts. These factors may all potentially influence the level of evidence of the outcomes. In the future, it will be necessary to conduct more rigorously designed, large-sample, multicenter, high-quality RCTs to further validate the clinical efficacy of PFBN.

## Conclusion

In conclusion, PFBN, PFNA, and InterTAN all yield satisfactory therapeutic outcomes in the treatment of intertrochanteric femoral fractures. Moreover, there were no significant differences between PFBN and the other two treatment modalities in terms of surgical duration, postoperative Harris score, intraoperative blood loss, incision length, or hip range of motion. However, PFBN has advantages over control treatments in terms of postoperative weight-bearing time, fracture healing time, quality of fracture reduction, postoperative complication rates, hospital stay, and postoperative VAS score. Therefore, in the future, on the basis of the situation of patients and the experience of surgeons, we can weigh the unique advantages of PFBN and the relative consistency of other aspects to develop the most appropriate surgical strategy to achieve earlier fracture healing and early weight bearing and reduce the occurrence of complications.

Nevertheless, the conclusions of this study still require further validation through more rigorously designed, large-sample, multicenter, high-quality RCTs to achieve more reliable results.

## Supplemental Information

10.7717/peerj.20801/supp-1 Supplemental Information 1 Meta-analysis results of p ostoperative hip Harris score In terms of the postoperative Harris score, although the scores of both control groups were lower than those of the PFBN group, indicating that PFBN had an advantage, this difference was not statistically significant

10.7717/peerj.20801/supp-2 Supplemental Information 2 Meta-analysis results of postoperative VAS score Compared with PFNA, PFBN has significant therapeutic advantages in terms of postoperative VAS score. However, compared with InterTAN, PFBN showed no significant statistical difference.

10.7717/peerj.20801/supp-3 Supplemental Information 3 Meta-analysis results of the hospital stay Compared with PFNA and InterTAN, the PFBN group had relatively shorter hospital stays, but the difference was not statistically significant. Overall, however, the advantage of PFBN has certain statistical significance (*P* = 0.05, 95% CI [−0.33–0.00]).

10.7717/peerj.20801/supp-4 Supplemental Information 4 Meta-analysis results of the intraoperative blood loss Compared with PFNA, PFBN has obvious advantages in terms of postoperative and intraoperative blood loss. However, compared with InterTAN, PFBN has no obvious advantage and there is no statistical difference.

10.7717/peerj.20801/supp-5 Supplemental Information 5 Meta-analysis results of the operation duration Whether compared with the PFNA group or the InterTAN group, the operation time of the PFBN group was relatively longer, but the difference was not statistically significant.

10.7717/peerj.20801/supp-6 Supplemental Information 6 The funnel plot of operation duration A symmetrical funnel shape indicating that the risk of publication bias in the included studies regarding the duration of surgery is not significant.

10.7717/peerj.20801/supp-7 Supplemental Information 7 Search strategy

10.7717/peerj.20801/supp-8 Supplemental Information 8 Raw Data

10.7717/peerj.20801/supp-9 Supplemental Information 9 PRISMA checklist

## Abbreviations

PFBN: Proximal femur bionic nail
PFNA: Proximal femoral nail anti-rotation
IFF: Intertrochanteric femoral fracture
CNKI: China National Knowledge Infrastructure
RevMan 5.4: Review Manager 5.4
VAS: Visual analog scale
SMD: Standardized mean difference
CI: Confidence interval
RR: Relative risk
PRISMA: Preferred Reporting Items for Systematic Reviews and Meta-Analyses
NOS: Newcastle-Ottawa Quality Assessment Scale
RCTs: Randomized controlled trials.

## Additional Information and Declarations

### Data Availability

The following information was supplied regarding data availability: This is a systematic review/meta-analysis.

## References

[ref-1] Atzmon R, Drexler M, Ohana N, Nyska M, Palmanovich E, Dubin J (2022). The effect of postoperative weight-bearing status on mortality rate following proximal femoral fractures surgery. Archives of Orthopaedic and Trauma Surgery.

[ref-2] Chang SM, Hou ZY, Hu SJ, Du SC (2020). Intertrochanteric femur fracture treatment in Asia: what we know and what the world can learn. Orthopedic Clinics of North America.

[ref-3] Chen P, Fan Z, Xu N, Wang H (2023). A biomechanical investigation of a novel intramedullary nail used to salvage failed internal fixations in intertrochanteric fractures. Journal of Orthopaedic Surgery and Research.

[ref-4] Chen ZX, Hu CY, Zhen ZH, Jiang HX, Gao MM, Wu BW, Huang GF, Ding ZQ (2020b). Effectiveness of proximal femoral nail anti-rotation combined with minimally invasive percutaneous plate osteosynthesis *versus* Intertan intramedullary nail fixation in treatment of intertrochanteric fracture with incomplete lateral wall. Chinese Journal of Reparative and Reconstructive Surgery.

[ref-5] Chen X, Tang M, Zhang X, Zhang Y, Wang Y, Xiong C, Ji Y, Wang Y, Zhang D (2024). A novel internal fixation design for the treatment of AO/OTA-31A3.3 intertrochanteric fractures: finite element analysis. Orthopaedic Surgery.

[ref-6] Chen HF, Yang DS, Leng JS, Chen P, Li Z, Wang PR, Wu GL, Yu GR (2020a). Bone cement-enhanced proximal femoral nail anti-rotation for treatment of severe osteoporotic intertrochanteric fracture. Chinese Journal of Orthopaedic Trauma.

[ref-7] Cheng X, Yang Y, Zhu J, Li G, Chen W, Wang J, Zhang Q, Zhang Y (2023). Finite element analysis of basicervical femoral neck fracture treated with proximal femoral bionic nail. Journal of Orthopaedic Surgery and Research.

[ref-8] Cochrane Collaboration (2020). https://china.cochrane.org/resources/cochrane-resources/software.

[ref-9] Fragility Fracture Network-China, Academic Committee of Orthopeadic Trauma, Bone and Joint Branch of Chinese Geriatrics Society, Bethune Alliance of Enhanced Recovery After Surgery in Orthopeadics, Orthopeadic Trauma Professional Committee, Bethune Public Welfare Foundation, Joint Surgery Professional Committee, Bethune Public Welfare Foundation, Orthopaedic Trauma Group, Branch of Enhanced Recovery After Surgery, China International Exchange and Promotion Association for Medicine and Health Care (2020). Guidelines for management of geriatric femoral intertrochanteric fractures. Chinese Journal of Traumatology.

[ref-10] Fu H, Hu L, Zou F, Liao X, Zheng Y, Jin P, Jia J, Xu J (2024). A comparative study of the early postoperative outcome of three intramedullary fixation modalities in the treatment of intertrochanteric fractures of the femur in the elderly. Journal of Musculoskeletal Neuronal Interactions.

[ref-11] Fu HP, Zhou YF, Jia JJ, Jing PC, Xu JC (2023). Early efficacy comparison of PFBN, InterTan and PFNA in the treatment of elderly intertrochanteric femoral fractures. Chinese Journal of Bone and Joint Injury.

[ref-12] Heydar AM, Kıyak G (2024). Posterolateral wall integrity in reverse oblique intertrochanteric fracture fixation: a new perspective in evaluation. Ulusal Travma ve Acil Cerrahi Dergisi.

[ref-13] Jia X, Qiang M, Zhang K, Han Q, Wu Y, Chen Y (2021). Influence of timing of postoperative weight-bearing on implant failure rate among older patients with intertrochanteric hip fractures: a propensity score matching cohort study. Frontiers in Medicine.

[ref-14] Jia C, Yu WZ, Wang YC, Qian ZP, He ZN, Zhu WK (2023). Proximal femoral bionic intramedullary nail *versus* InterTAN intramedullary nail in the treatment of intertrochanteric femoral fractures in elderly patients. Journal of Clinical Medicine and Practice.

[ref-15] Jin LK, Li H, Zhang J, Dong YX, Qi YF (2024). Clinical observation of different intramedullary fixation methods for the treatment of intertrochanteric fracture. China Journal of Orthopaedics and Traumatology.

[ref-16] Li M, Li ZR, Li JT, Lei MX, Su XY, Wang GQ, Zhang H, Xu GX, Yin P, Zhang LC, Tang PF (2019). Three-dimensional mapping of intertro-chanteric fracture lines. Chinese Medical Journal.

[ref-17] Li ZT, Yin J, Xiao W, Shi SY, Che LX, Sun JG (2022). Proximal femoral biomimetic intramedullary nail *versus* proximal femoral anti-rotation intramedullary nail in treatment of senile osteoporotic intertrochanteric fractures. China Journal of Orthopaedics and Traumatology.

[ref-18] Lin D, Chen CQ, Wang S, Xie CW, Guo ZS, Cui XH, Zhao Z (2022). Early efficacy comparison of proximal femoral bionic nail and proximal femoral nail anti-rotation in the treatment of intertrochanteric fracture in the elderly. Chinese Journal of Traumatology.

[ref-19] Ling F, Zhang W, Chang R, Zhang Z, Liu HS (2023). Proximal femoral bionic nail *versus* proximal femoral nail anti-rotation in treatment of femoral intertrochanteric fracture: a comparison of short-term efficacy. Chinese Journal of Orthopaedic Trauma.

[ref-20] Liu PY, Huang H, Xiao J, Peng FP, Ye JG, Zhang L, Fu NX (2023). Clinical study of proximal femoral biomimetic intramedullary nail on the treatment of femoral intertrochanteric fracture complicated with osteoporosis. Chinese Journal of Traditional Medical Traumatology & Orthopedics.

[ref-21] Liu P, Wu X, Shi H, Liu R, Shu H, Gong J, Yang Y, Sun Q, Wu J, Nie X, Cai M (2015). Intramedullary *versus* extramedullary fixation in the management of subtrochanteric femur fractures: a meta-analysis. Clinical Interventions in Aging.

[ref-22] Long ZM, Li ZZ (2022). Comparison of the stability of proximal femur bionic nail and traditional proximal femoral nail anti-rotation in the treatment of unstable intertrochanteric fracture. Reflex Rehabilitation Medicine.

[ref-23] Lu GL, Li SJ, Li WX (2022). Biomechanical study of extramedullary and intramedullary fixation in the treatment of unstable intertrochanteric reversed-tilt fractures of the femur. Annals of Translational Medicine.

[ref-24] Mavrogenis AF, Panagopoulos GN, Megaloikonomos PD, Igoumenou VG, Galanopoulos I, Vottis CT, Karabinas P, Koulouvaris P, Kontogeorgakos VA, Vlamis J, Papagelopoulos PJ (2016). Complications after hip nailing for fractures. Orthopedics.

[ref-25] Nie SB, Zhang W, Zhang LC, Zhang W, Tang PF (2021). Progress in the study of risk factors for internal fixation failure after intertrochanteric fracture. Chinese Journal of Traumatology.

[ref-26] Page MJ, McKenzie JE, Bossuyt PM, Boutron I, Hoffmann TC, Mulrow CD, Shamseer L, Tetzlaff JM, Akl EA, Brennan SE, Chou R, Glanville J, Grimshaw JM, Hróbjartsson A, Lalu MM, Li T, Loder EW, Mayo-Wilson E, McDonald S, McGuinness LA, Stewart LA, Thomas J, Tricco AC, Welch VA, Whiting P, Moher D (2021). The PRISMA 2020 statement: an updated guideline for reporting systematic reviews. BMJ.

[ref-27] Shi Z, Qiang M, Jia X, Zhang K, Chen Y (2021). Association of the lateral wall integrity with clinical outcomes in older patients with intertrochanteric hip fractures treated with the proximal femoral nail anti-rotation-Asia. International Orthopaedics.

[ref-28] Stang A (2010). Critical evaluation of the Newcastle–Ottawa scale for the assessment of the quality of nonrandomized studies in meta-analyses. European Journal of Epidemiology.

[ref-29] Sun ZH, Chen D, Chu KW, Shi Y, Hong B, Chen Y, Liu L (2024). Comparison of clinical data between the proximal femoral bionic nail (PFBN) and hip replacement for the treatment of femoral intertrochanteric fracture. European Review for Medical and Pharmacological Sciences.

[ref-30] Veronese N, Maggi S (2018). Epidemiology and social costs of hip fracture. Injury.

[ref-31] Wan Y, Chen SD, Luo YF, Yang ZH, Zhao ZH, Huang WL, Deng J (2024). Two types of proximal femoral intramedullary nail fixation for unstable intertrochanteric fractures of the femur. Chinese Journal of Orthopaedics.

[ref-32] Wang Y, Chen W, Zhang L, Xiong C, Zhang X, Yu K, Ju J, Chen X, Zhang D, Zhang Y (2022c). Finite element analysis of proximal femur bionic nail (PFBN) compared with proximal femoral nail anti-rotation and InterTAN in treatment of intertrochanteric fractures. Orthopaedic Surgery.

[ref-33] Wang T, Guo J, Long Y, Hou Z (2022b). Incidence and risk factors of mortality in nonagenarians and centenarians after intertrochanteric fracture: 2-year follow-up. Clinical Interventions in Aging.

[ref-34] Wang H, Yang W, Ding K, Zhu Y, Zhang Y, Ren C, Zhao K, Zhang Q, Chen W, Zhang Y (2022a). Biomechanical study on the stability and strain conduction of intertrochanteric fracture fixed with proximal femoral nail anti-rotation *versus* triangular supporting intramedullary nail. International Orthopaedics.

[ref-35] Wang YC, Yu WZ, Wu GM, Zhang WF, Pan JL, Zhu WK, He ZN, Xu P, Jia C (2023). Comparison of two kinds of proximal femoral intramedullary nail for intertrochanteric femoral fractures in the elderly. Orthopedic Journal of China.

[ref-36] Wu YX, Lin ZH, Huang HB, Tang QC (2023). Comparison of effect of three fixation methods in the treatment of intertrochanteric fracture of femur in the elderly. Chinese Journal of Modern Drug Application.

[ref-37] Wu YK, Wang RQ, Ning SP, Yang RJ, Zhu XF (2022). Risk factors of proximal femoral nail anti-rotation failure for femoral intertrochanteric fractures. Orthopedic Journal of China.

[ref-38] Xie H, Xie L, Wang J, Chen C, Zhang C, Zheng W (2019). Intramedullary *versus* extramedullary fixation for the treatment of subtrochanteric fracture: a systematic review and meta-analysis. International Journal of Surgery.

[ref-39] Yang DS, Wang Q, Luan ZH, Lin JS, Chen P, Chen XD, Yuan DT, Zhen XZ, Wang JQ (2023). Effectiveness of proximal femur bionic nail for intertrochanteric fracture in the elderly. Chinese Journal of Reparative and Reconstructive Surgery.

[ref-40] Zeelenberg ML, Nugteren LHT, Plaisier AC, Loggers SAI, Joosse P, Den Hartog D, Verhofstad MHJ, Van Lieshout EMM, STABLE-HIP Study Group (2023). Extramedullary *versus* intramedullary fixation of stable trochanteric femoral fractures: a systematic review and meta-analysis. Archives of Orthopaedic and Trauma Surgery.

[ref-41] Zeelenberg ML, Plaisier AC, Nugteren LHT, Loggers SAI, Joosse P, Verhofstad MHJ, Den Hartog D, Van Lieshout EMM, STABLE-HIP Study Group (2024). Extramedullary *versus* intramedullary fixation of unstable trochanteric femoral fractures (AO type 31-A2): a systematic review and meta-analysis. Archives of Orthopaedic and Trauma Surgery.

[ref-42] Zhang D, Yu K, Zhao X (2019). Proximal femur bionic nail system with anti-rotational and bionic reconstruction support. https://patents.google.com/patent/CN205658941U/en.

[ref-43] Zhang DY (2020). The past and future of internal fixation for femoral intertrochanteric fractures: a perspective from lever fulcrum reconstruction theory. Chinese Journal of Orthopaedic Trauma.

[ref-44] Zhang DY, Yu K, Yang J, Zhao XT, Zhang XM, Wang YH, Ju JB (2020). Lever-pivot balance: a neodoxy on treatment for intertrochanteric femoral fractures. Chinese Journal of Traumatology.

[ref-45] Zhang RB, Peng WJ (2022). Analysis of clinical effect of proximal femoral bionic nail in the treatment of unstable intertrochanteric fracture of the femur in elderly patients. Chinese Practice of Medicine.

[ref-46] Zhang W, Chen H, Li JT, Qi L, Tang PF (2021a). Efficacy of triangular mechanical reconstruction for treatment of failed fixation of intertrochanteric factures. Chinese Journal of Traumatology.

[ref-47] Zhang WQ, Sun J, Liu CY, Zhao HY, Sun YF (2018). Comparing the intramedullary nail and extramedullary fixation in treatment of unstable intertrochanteric fractures. Scientific Reports.

[ref-48] Zhang XM, Yu K, Wang YH, Yang J, Zhao XT, Ju JB, Zhang DY (2021b). Analysis of characteristics and causes of postoperative invalid fixation failures of femoral intertrochanteric fractures. Chinese Journal of Traumatology.

[ref-49] Zhang Y, Chen W, Zhang Q (2010). A triangular-supported intramedullary nail for femoral neck and intertrochanteric fractures. https://patents.google.com/patent/CN201524132U/en.

[ref-50] Zhang Y, Zhang YC, Zhang DY (2024). Comparison of proximal femoral bionic nail and proximal femoral nail anti-rotation in fixation of intertrochanteric fractures of the femur in the aged patients. Chinese Journal of Orthopaedic Trauma.

[ref-51] Zhang YZ, Wang HC, Chen Z, Zhu YB, Ding K, Hou ZY, Zhang Q, Wang J (2021c). Triangular supporting fixation: an innovative surgical approach for intertrochanteric fractures of the femur–Evidence from a biomechanical study. Chinese Journal of Orthopaedic Trauma.

[ref-52] Zhu YB, Chen W, Ye DD, Zhang Q, Lv HZ, Zheng ZL, Zhang YZ (2021a). Proximal Femur N triangle theory and the design concept of proximal femur bionic nail (PFNB). Chinese Journal of Geriatric Orthopaedics & Rehabilitation (Electronic Edition).

[ref-53] Zhu YB, Ding K, Li YL, Wang HC, Chen W, Hou ZY, Zhang Q, Wang J, Zhang YZ (2021b). Biomechanical comparison of triangle supporting fixation system and Gamma nail fixation in the treatment of intertrochanteric fractures of the femur: finite element analysis. Chinese Journal of Orthopaedics.

