# Supplementary figures and images for: Clinical effects and safety of proximal femur bionic nail versus proximal femoral nail anti-rotation or InterTAN for the treatment of intertrochanteric femoral fracture: a systematic review and meta-analysis
Source: PeerJ. 2026 Feb 16;14:e20801. doi: 10.7717/peerj.20801 (PMC12919315; doi:10.7717/peerj.20801)

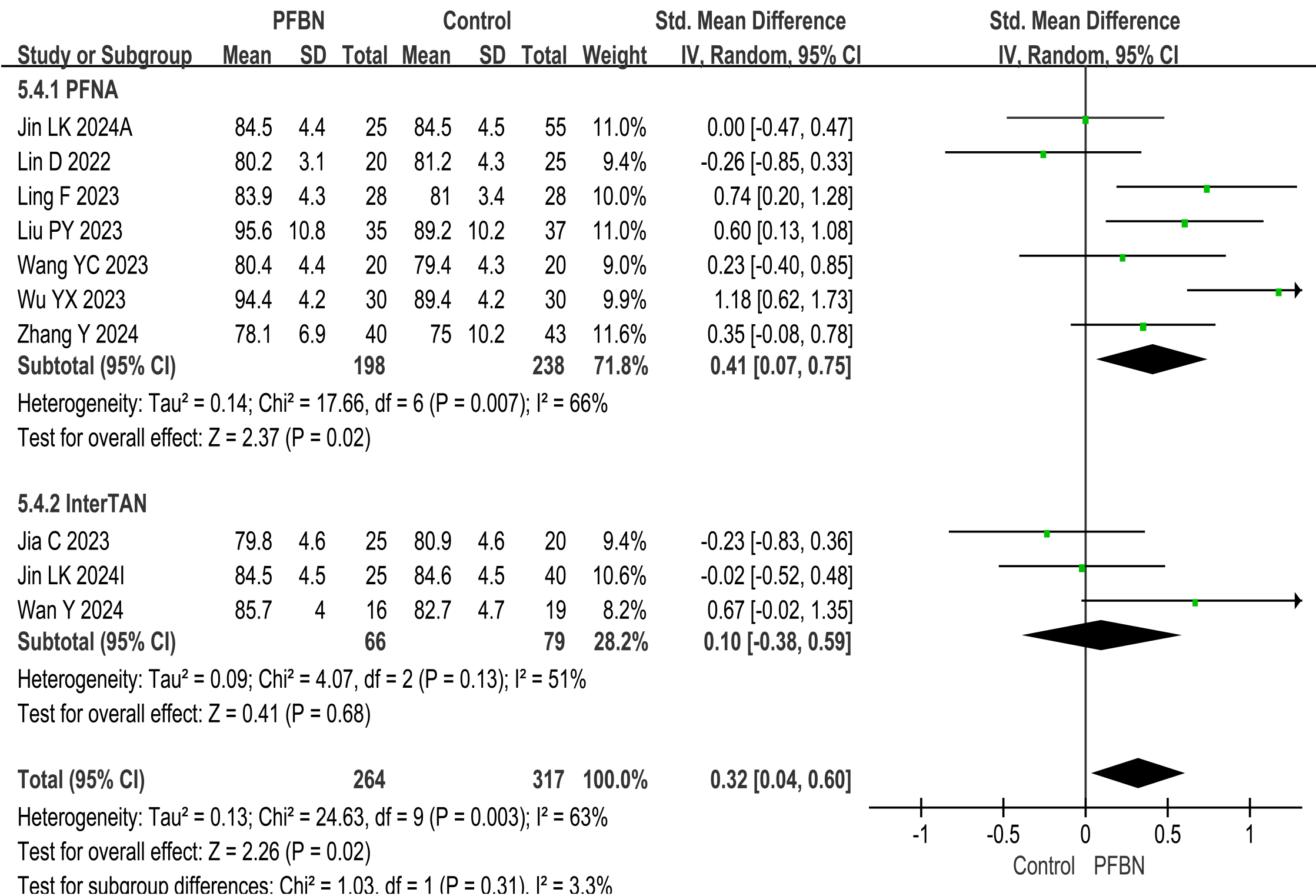

Supplement: Supplemental Information 1 — In terms of the postoperative Harris score, although the scores of both control groups were lower than those of the PFBN group, indicating that PFBN had an advantage, this difference was not statistically significant [file peerj-14-20801-s001.pdf]

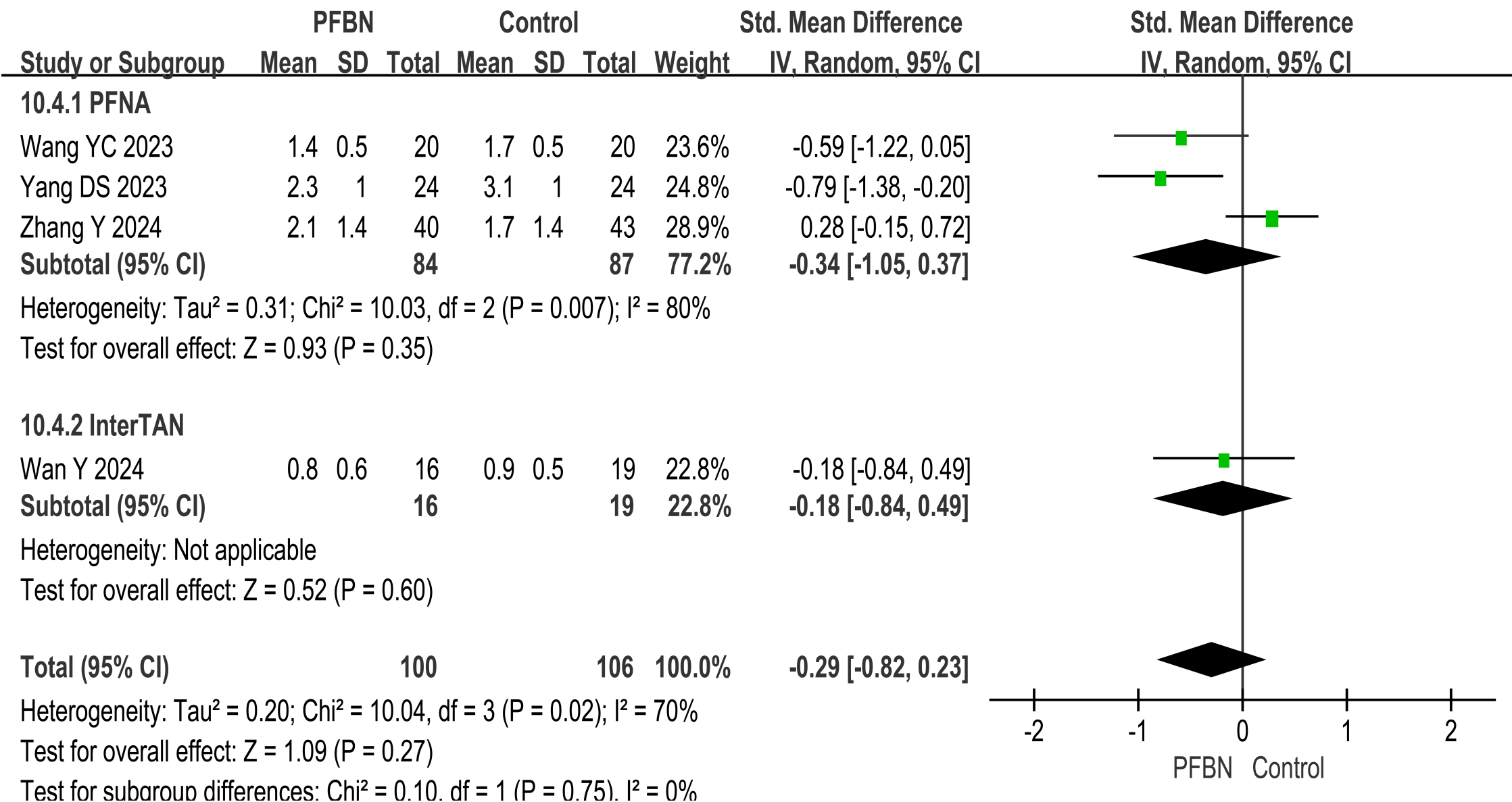

Supplement: Supplemental Information 2 — Compared with PFNA, PFBN has significant therapeutic advantages in terms of postoperative VAS score. However, compared with InterTAN, PFBN showed no significant statistical difference. [file peerj-14-20801-s002.pdf]

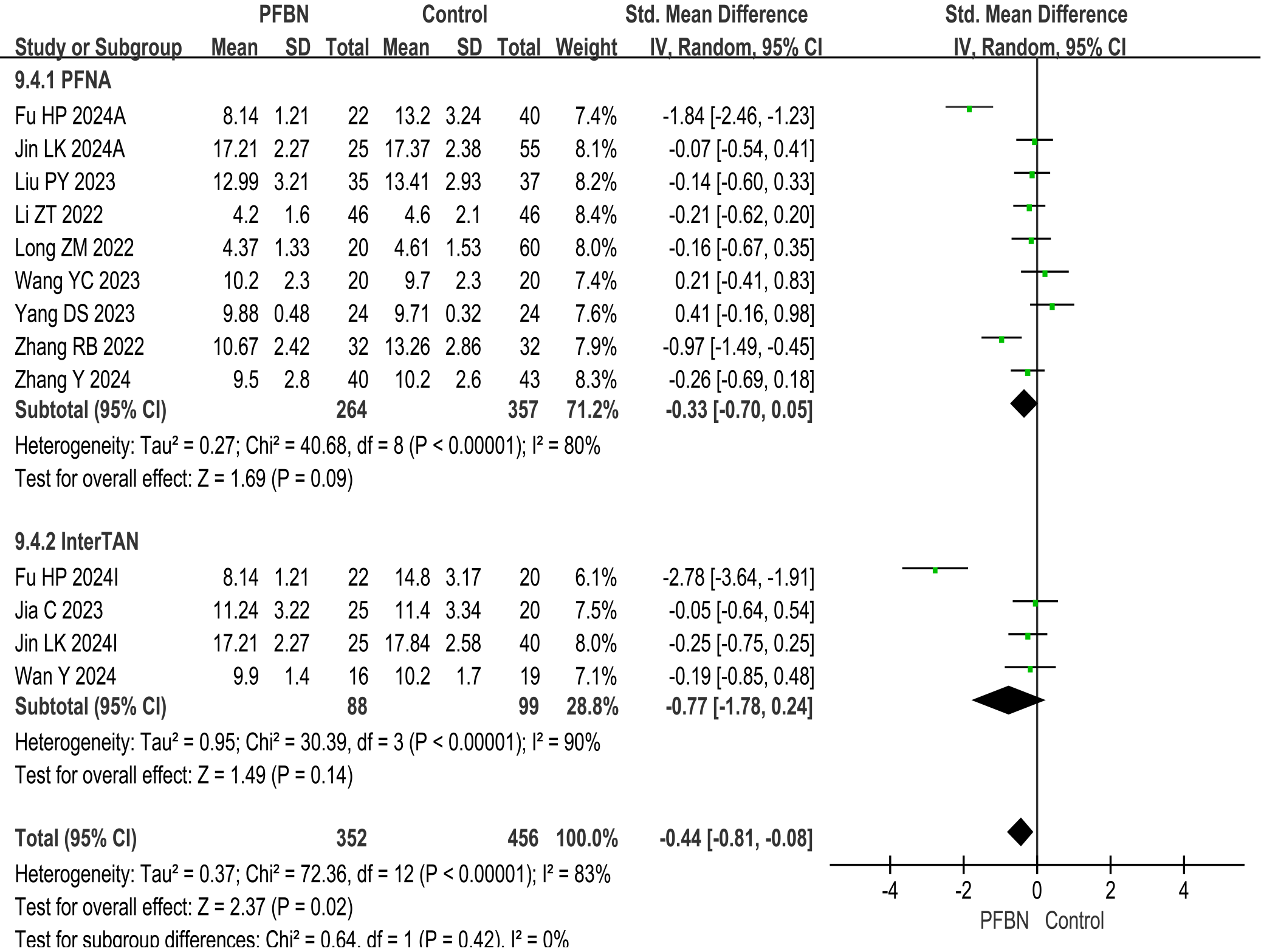

Supplement: Supplemental Information 3 — Compared with PFNA and InterTAN, the PFBN group had relatively shorter hospital stays, but the difference was not statistically significant. Overall, however, the advantage of PFBN has certain statistical significance (P = 0.05, 95% CI [−0.33–0.00]). [file peerj-14-20801-s003.pdf]

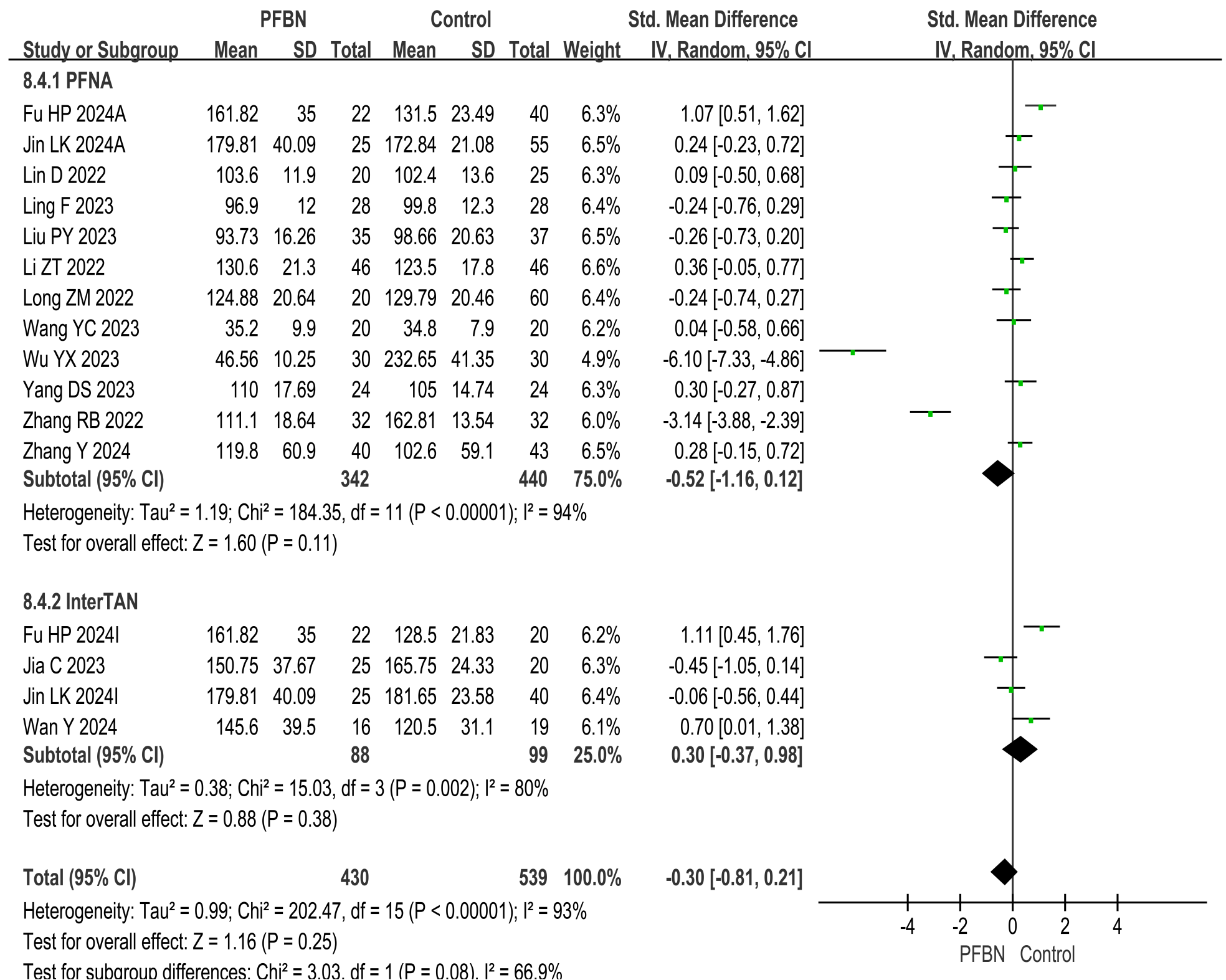

Supplement: Supplemental Information 4 — Compared with PFNA, PFBN has obvious advantages in terms of postoperative and intraoperative blood loss. However, compared with InterTAN, PFBN has no obvious advantage and there is no statistical difference. [file peerj-14-20801-s004.pdf]

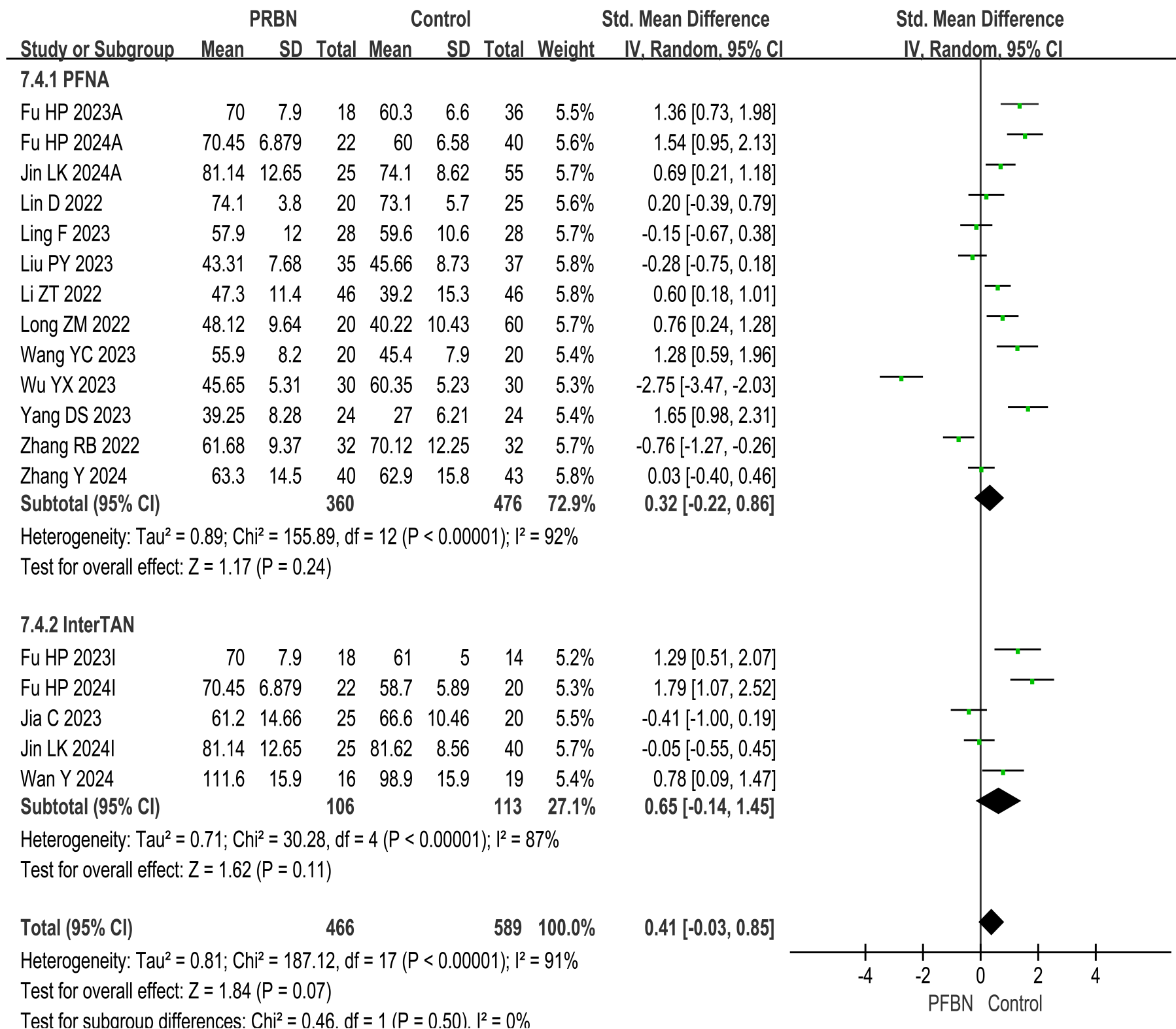

Supplement: Supplemental Information 5 — Whether compared with the PFNA group or the InterTAN group, the operation time of the PFBN group was relatively longer, but the difference was not statistically significant. [file peerj-14-20801-s005.pdf]

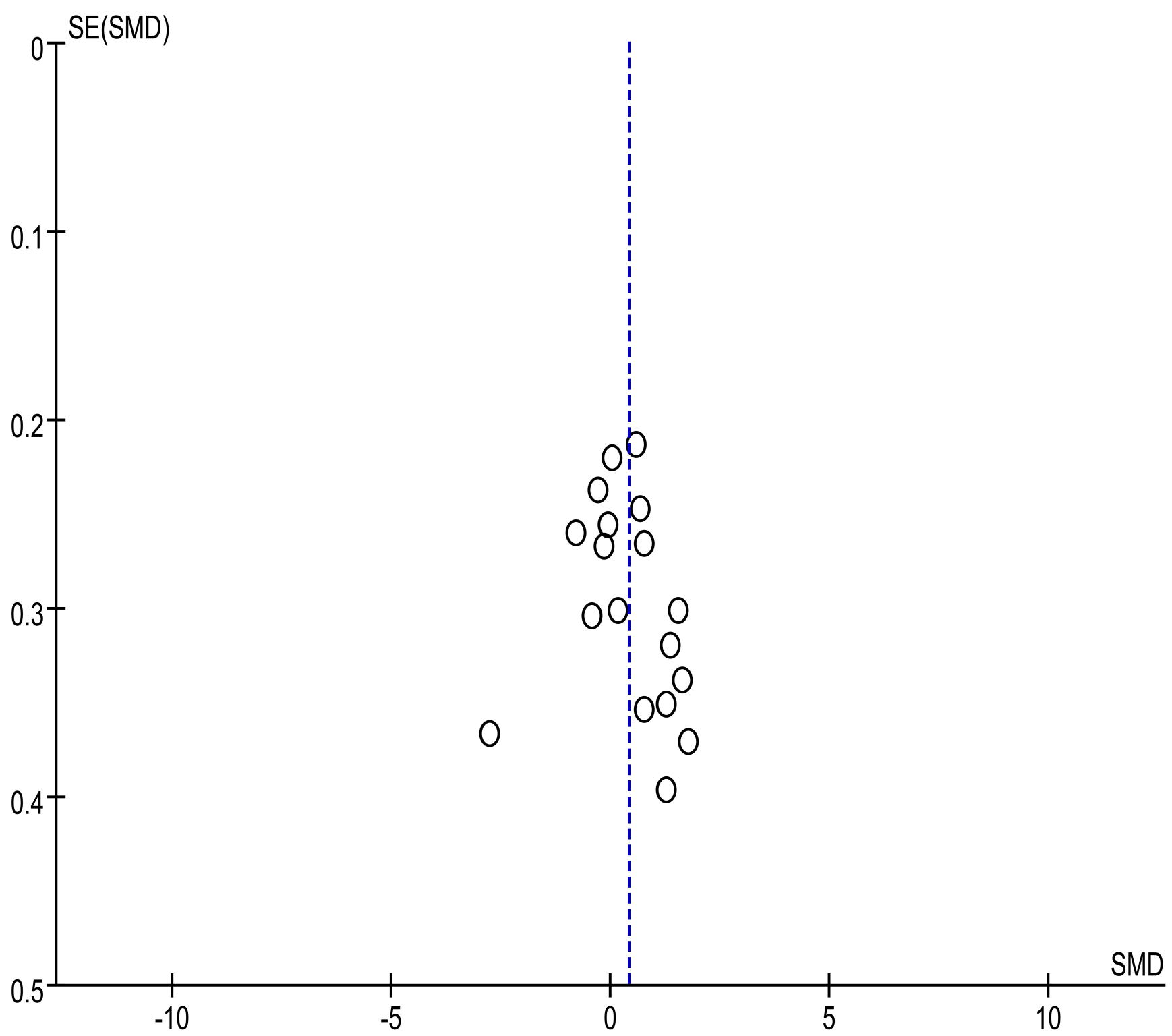

Supplement: Supplemental Information 6 — A symmetrical funnel shape indicating that the risk of publication bias in the included studies regarding the duration of surgery is not significant. [file peerj-14-20801-s006.pdf]
