# Supplementary material for: Clinical effects and safety of proximal femur bionic nail versus proximal femoral nail anti-rotation or InterTAN for the treatment of intertrochanteric femoral fracture: a systematic review and meta-analysis
Source: PeerJ. 2026 Feb 16;14:e20801. doi: 10.7717/peerj.20801 (PMC12919315; doi:10.7717/peerj.20801)
Supplement: Supplemental Information 7 [file peerj-14-20801-s007.pdf]

知网(CNKI)：

（主题：股骨近端仿生髓内钉）OR（主题：PFBN） 24

Translations:(Topic: Proximal femur bionic nail) OR (Topic: PFBN)

维普(VIP)：

(股骨近端仿生髓内钉+PFBN+仿生髓内钉) 43

Translations:(Proximal femur bionic nail+ PFBN+ Bionic intramedullary nail)

万方(Wanfang)：

(主题=股骨近端仿生髓内钉) OR (主题=PFBN) 80

Translations: (Topic=Proximal femur bionic nail) OR (Topic=PFBN)

Pubmed：

(proximal femur bionic nail) OR (PFBN) 32

Web of science:

(ALL=(proximal femur bionic nail)) OR ALL=(PFBN) 30

EMBASE：

proximal AND femur AND bionic AND nail OR pfbn 20

Cochrane:

proximal femur bionic nail in All Text OR PFBN in All Text 2
